# Prominent PD-L1-positive M2 macrophage infiltration in gastric cancer with hyper-progression after anti-PD-1 therapy: A case report

**DOI:** 10.1097/MD.0000000000025773

**Published:** 2021-05-14

**Authors:** Kyoko Yamaguchi, Kenji Tsuchihashi, Kunihiro Tsuji, Yosuke Kito, Kenro Tanoue, Hirofumi Ohmura, Mamoru Ito, Taichi Isobe, Hiroshi Ariyama, Hitoshi Kusaba, Koichi Akashi, Eishi Baba

**Affiliations:** a Department of Medicine and Biosystemic Science, Graduate School of Medical Sciences, Kyushu University, Fukuoka; b Department of Medical Oncology, Ishikawa Prefectural Central Hospital, Ishikawa; c Department of Oncology and Social Medicine, Graduate School of Medical Sciences, Kyushu University, Fukuoka, Japan.

**Keywords:** hyper-progressive disease, nivolumab, type 2 macrophage

## Abstract

**Rationale:**

Anti-PD-1 antibody is the standard therapy for treatment-resistant gastric cancer, but only a limited number of patients respond. Additionally, cases of hyper-progressive disease (HPD) in which tumor growth accelerates after anti-PD-1 antibody administration have been reported; however, the biological mechanism has not been elucidated.

**Patient concerns:**

In the present case, metastatic gastric cancer was treated with the anti-PD-1 antibody, nivolumab, as third-line treatment.

**Diagnosis:**

After the initiation of nivolumab therapy, a rapidly enlarging para-aortic lymph nodes were observed leading to the diagnosis of HPD.

**Interventions:**

Multiplex immunohistochemistry was used to examine immune cells infiltrating in the primary tumor and in liver metastasis which were obtained before nivolumab treatment, and in lymph node metastasis which presented with HPD after nivolumab therapy.

**Outcomes:**

In the primary tumor, helper T (Th) cells, cytotoxic T lymphocytes (CTLs), regulatory T (Treg) cells, and PD-L1-negative macrophages were observed. On the other hand, in metastatic lymph nodes presenting with HPD, PD-L1-positive macrophages prominently increased, while Treg cells, CTLs, and Th cells decreased. PD-L1 expression was not observed in gastric cancer cells among the three specimens.

**Lessons:**

The findings suggest the possibility that PD-L1-positive M2 macrophage might contribute to acceleration of tumor growth with anti-PD-1 therapy in the present case.

## 1 Introduction

Anti-PD-1 antibody monotherapy with nivolumab prolonged overall survival compared with placebo in late-line advanced gastric cancer (AGC) patients in a phase 3 trial.^[1]^ The overall response rate and disease control rate were 11.2% and 40.3%, respectively, whereas progressive disease was observed in 46% of patients even after nivolumab-administration, suggesting that these tumors were primary resistant.^[1]^ Similar to the results of immune checkpoint inhibitors (ICIs) for other cancers, a response is observed in a limited patient population. The biological mechanisms of primary resistance to ICIs are thought to be based on 2 categories: intrinsic mechanisms of the tumor cell, and extrinsic mechanisms of the tumor microenvironment.^[2]^ Intrinsic mechanisms include low tumor mutation burden associated with fewer neoantigens that tumor-specific T cells can target and a decreased efficiency of antigen-presenting mechanisms of tumor cells missing MHC molecule. Extrinsic mechanisms are closely associated with the tumor microenvironment including a lack of effective cytotoxic T lymphocyte (CTL) infiltration and an increased number of immune suppressive cells such as type 2 macrophages (M2 macrophages), regulatory T (Treg) cells, and myeloid-derived suppressor cells. In cases of less tumor-specific CTL infiltration but increased immune suppressive cells, tumor eradication is thought to be difficult, even if an ICI is administered. Therefore, reveling immune cell profile in tumor tissue might predict the effectiveness of ICI therapy.

Recently, cases of accelerated tumor growth after ICI administration have been reported as hyper-progressive disease (HPD). HPD has been reported in various types of cancers such as non-small cell lung cancer (NSCLC), head and neck squamous cell carcinoma, urothelial bladder carcinoma, melanoma, and gastric cancer.^[3–7]^ The overall survival of HPD patients is shorter compared with patients without HPD.^[3]^ HPD is described as a phenomenon with a higher increment in tumor volume during the period from prior to ICI administration to after ICI administration compared to the period from the latest assessment of tumor size during the previous therapy regimen (i.e., prior to ICI administration). Although no consensus definition of HPD has been established, the following definitions are mainly used^[8]^:

1. Time to treatment failure, the time between the beginning of ICI therapy to interruption for any reason is within 2 months (time to treatment failure ≤2 months)
2. tumor growth rate, in contrast with the pretherapy images, the patient's tumor volume is doubled or increased (tumor growth rate ≥2)
3. TGK (tumor growth kinetics), objective lesion changes in unit intervals determined by evaluation of the largest diameters according to RECIST (TGK ≥2)

More specifically, TGK_PRE_ was calculated as the difference in the sum of the largest diameters of the target lesions per unit of time between prebaseline and baseline imaging: (S_0_ – S_PRE_)/(T_0_ – T_PRE_). Similarly, TGK_POST_ was calculated as (S_POST_ – S_0_)/(T_POST_ – T_0_). In the present study, the TGK ratio (TGKR) was defined as TGK_POST_/TGK_PRE_.^[7]^

The definite biological mechanisms of HPD following ICI treatment have not been elucidated, but acceleration of treatment resistance in both intrinsic and extrinsic mechanisms is suspected.^[8]^ The incidence of HPD following ICI treatment ranges from 4% to 29% in previous studies.^[9]^ As for gastric cancer, some retrospective analyses showed that the incidence of HPD is 21% to 29% in patients treated with anti-PD-1 therapy,^[7,10]^ in which the biological mechanisms of HPD in AGC patients have not been examined. Therefore, in the present study, we comprehensively measured immune cells in tumor tissues before and after anti-PD-1 therapy in an AGC patient with HPD. The present study showed a significant increase in PD-L1-positive macrophages in AGC metastatic tissue with significant tumor growth after anti-PD-1 therapy.

## 2 Materials and methods

### 2.1 Multiplex immunohistochemical staining

Sections were produced from formalin-fixed paraffin–embedded (FFPE) blocks of surgically resected primary tumor and liver metastatic tumor, and from a metastatic lymph node obtained by fine needle biopsy. The sections were deparaffinized in xylene and rehydrated in ethanol. Antigen retrieval was performed in Tris EDTA buffer (Antigen Unmasking Solution, High pH, Vector Laboratories, Burlingame, CA) with microwave treatment (MWT) for 20 minutes. Endogenous peroxidases were blocked using 3% hydrogen peroxide in distilled water for 5 minutes. Protein blocking was performed using Protein Block Serum-Free (Agilent Technologies, Santa Clara, CA). Multiplex immunohistochemical staining was conducted using the Opal seven-color IHC Kit (Perkin Elmer, Waltham, CA). Sections were incubated with a primary antibody for CD8 (1:50, clone C8/144B, Agilent Technologies) for 30 minutes, and then incubated using EnVision+ Dual Link kit (Agilent Technologies) for 10 minutes, followed by visualization using Opal520 TSA (1:50, Perkin Elmer) for another 10 minutes. After that, MWT was repeated to remove the antibody and TSA complex. Using this Opal staining method, all samples were stained sequentially with a primary antibody for PD-L1 (1:200, clone E1L3N, Cell Signaling, Danvers, MA) visualized with Opal540 TSA (1:50), FoxP3 (1:50, clone SP97, Abcam, Cambridge, UK) visualized with Opal570 TSA (1:50), CD3 (1:50, clone F7.2.38, Agilent Technologies) visualized with Opal620 TSA (1:50), CD163 (1:200, clone 10D6, Leica Biosystems, Wetzlar, Germany) visualized with Opal650 TSA (1:50), and Cytokeratin (1:100, clone AE1/AE3, Invitrogen, Carlsbad, CA) visualized with Opal690. TSA-stained slides were finished with MWT, counterstained with DAPI for 5 minutes, and coverslipped using VectaShield Hardset mounting media (Vector Laboratories) (Fig. 2).

**Figure 1:**
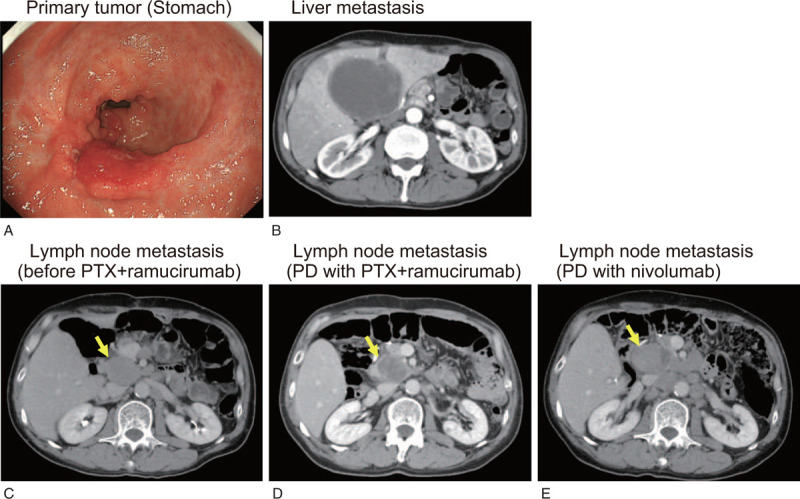
Clinical images during course of treatment. (A) Upper gastrointestinal endoscopy image at diagnosis. A Borrmann type 3 tumor that protruded was located at the pyloric antrum. (B) A computed tomography (CT) image of liver metastasis. The tumor located at the liver S4 lesion increased in size, and its internal area with poor contrast was enlarged. This lesion was suspected to be complicated by an abscess. (C-E) CT images of lymph node metastasis at the time before paclitaxel (PTX) plus ramucirumab therapy (C), disease progression after PTX plus ramucirumab therapy (D), and disease progression after nivolumab therapy (E). Metastatic lymph nodes (yellow arrows) remarkably increased in size after nivolumab therapy (c-e).

**Figure 2:**
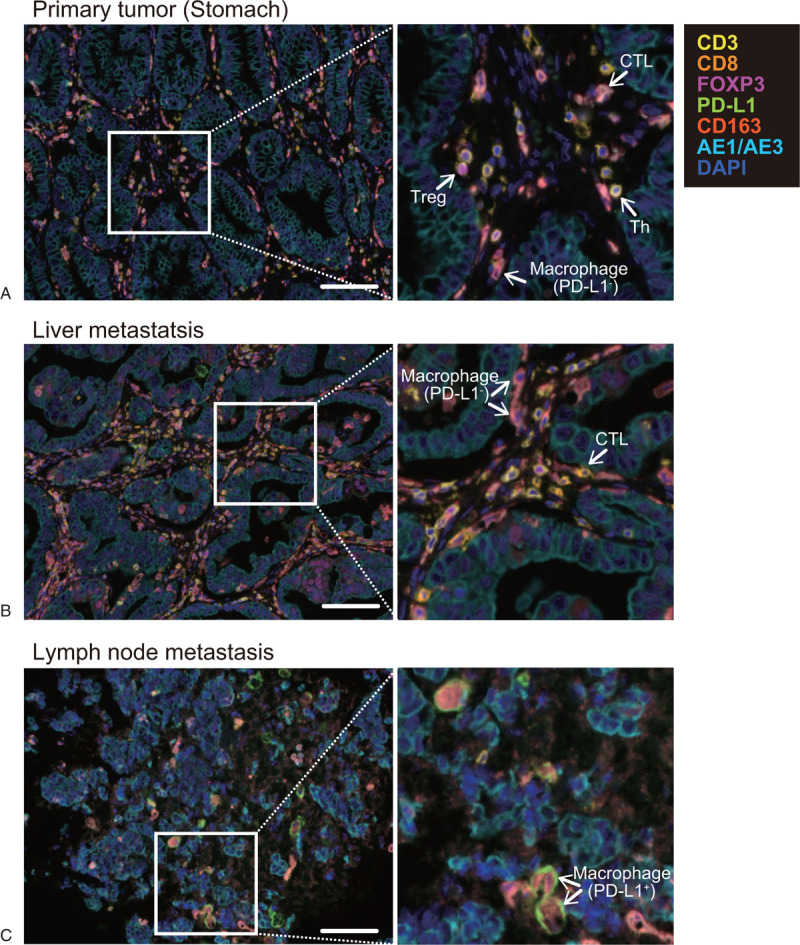
Representative images of the primary tumor (A), liver metastatic tumor (B), and metastatic lymph node (C). The sections were stained with CD3 (yellow), CD8 (orange), FoxP3 (magenta), PD-L1 (green), CD163 (salmon pink), AE1/AE3 (cyan), and DAPI (blue). The white arrows indicate immune cells: cytotoxic T lymphocytes (CTLs, CD3^+^CD8^+^), regulatory T (Treg) cells (CD3^+^CD8^−^FoxP3^+^), helper T (Th) cells (CD3^+^CD8^−^FoxP3^−^), and macrophages (CD3^−^CD163^+^). Magnification, ×200, Scale bar, 100 μm.

### 2.2 Multispectral image acquisition and analysis

**Figure 3:**
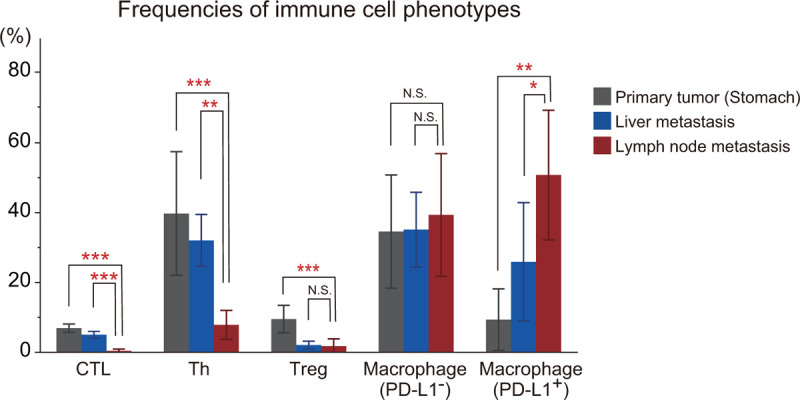
Frequencies of immune cell phenotypes in primary and metastatic tumors. Bar graphs represent the proportion of cytotoxic T lymphocytes (CTLs), helper T (Th) cells, regulatory T (Treg) cells, PD-L1-negative macrophages, and PD-L1-positive macrophages in the primary tumor, liver metastasis, and lymph node metastasis. Data represent the mean ± standard deviation of 3 independent experiments. ^∗∗∗^*P* < .001, ^∗∗^*P* < .01, ^∗^*P* < .05, N.S., not significant.

Sections stained with seven-plex fluorescence were scanned using the Mantra Quantitative Pathology Workstation (Perkin Elmer) at 200× magnification. Three different locations in the tumor area were scanned in 1 section. Images were used for the following analysis using inForm Advanced Image Analysis software (inForm version 2.3.0, Perkin Elmer). Multispectral images were unmixed using spectral libraries built from images of single-stained tissues for each marker. A selection of 3 representative multispectral images obtained from normal lymph node tissues, which were used as positive controls, was used to train the process of cell segmentation and cell phenotyping in the inForm software. All settings were saved in an algorithm to allow batch analysis of all multispectral images. Finally, the number of each cell phenotype was calculated for statistical analysis. Tumor tissue-infiltrating immune cells were defined by the pattern of fluorescence signals as follows: CTLs (CD3^+^CD8^+^), helper T (Th) cells (CD3^+^CD8^−^FoxP3^−^), Treg cells (CD3^+^CD8^−^FoxP3^+^), PD-L1-negative macrophages (CD3^−^CD163^+^PD-L1^−^), and PD-L1-positive macrophages (CD3^−^CD163^+^PD-L1^+^). Densities of immune cell phenotypes were calculated as the number of each phenotype divided by the total number of these 5 types of infiltrating immune cells in the image (Fig. 3).

### 2.3 Statistical analysis

Data were represented as the mean ± standard deviation of 3 independent experiments. Significance of differences in means between 2 groups was determined with the Student *t* test. *P* < .05 was considered statistically significant. All statistical analyses were carried out using JMP Pro version 14.2.0 software (SAS Institute Japan, Tokyo, Japan).

### 2.4 Ethics approval

The patient's family member has provided informed consent for publication of the case, because the patients were already deceased at the time of the analysis. The present study was conducted as part of a retrospective study using FFPE specimens at Kyushu University Hospital and Ishikawa Prefectural Central Hospital. The present study was approved by the institutional review board (IRB) of Kyushu University Hospital (No. 30–525) and Ishikawa Prefectural Central Hospital (No. 1308).

## 3 Case presentation

A 55-year-old male had suffered from epigastric discomfort since July 2013. The patient underwent upper gastrointestinal endoscopy and was diagnosed with type 3 AGC of the pyloric antrum of the stomach (T3N2bM0, cStage IIIC, according to the 7th edition of the UICC TNM classification of gastric cancer) (Fig. 1a). Histological diagnosis was well to poorly differentiated adenocarcinoma. Distal gastrectomy with D2 lymph node dissection for curative intent was performed in September 2013. The patient was given adjuvant chemotherapy with Tegafur/gimeracil/oteracil (S-1) for 1 year. In March 2016, metastatic liver tumors in S4 and S8 and enlarged hilar lymph nodes were confirmed by screening examination with computed tomography (CT). Combined chemotherapy with S-1 plus cisplatin (SP) was administered as first-line systemic chemotherapy. Immunohistochemistry of the primary tumor showed that HER2 expression was negative. After 1 cycle of SP therapy, the liver tumor in S4 increased, and an abscess developed inside the tumor (Fig. 1b). Percutaneous drainage was performed, but the abscess was difficult to manage. In May 2016, a partial liver resection of S4 and S8 was performed to control the infection. After the metastasectomy of the liver, 5 cycles of S-1 monotherapy and 17 cycles of paclitaxel (PTX) plus ramucirumab therapy were administered for the remaining hilar lymph node metastases and newly emerged para-aortic and mesenteric lymph node metastases. However, the metastatic lymph nodes gradually increased in size even after PTX plus ramucirumab therapy. The short axes of the largest para-aortic and hilar lymph nodes were 33.4 mm and 23.7 mm in the CT scan in September 2016 (Fig. 1c) before administration of PTX plus ramucirumab therapy, and 31.8 mm and 9.2 mm in November 2017 (Fig. 1d) after the last administration of PTX plus ramucirumab therapy (TGK_PRE_ was–1.9%). In December 2017, nivolumab monotherapy with 3 mg/kg biweekly intravenous administration was started as third-line therapy. However, at the first evaluation in 2 months after the initial administration of nivolumab, the size of the metastatic lymph nodes significantly increased to 35.3 mm and 22.8 mm in the CT scan (TGK_POST_ was 14.9%, |TGKR| was 7.8) (Fig. 1e). Although lymphadenopathy due to lymphatic metastasis of gastric cancer was suspected, the serum IgG4 level was high (195 mg/dL), and IgG4-related diseases could be also considered as a differential diagnosis. A fine needle biopsy was performed to confirm the diagnosis of a metastasis of gastric adenocarcinoma. The patient was diagnosed with HPD according to the rapid enlargement of metastatic lymph nodes after administration of nivolumab. Nivolumab monotherapy was terminated, followed by 2 cycles of capecitabine plus oxaliplatin as fourth-line therapy and 3 cycles of irinotecan monotherapy as fifth-line therapy. The metastatic lymph nodes grew further, and the patient died 8 months after nivolumab administration.

## 4 Results

**Figure 4:**
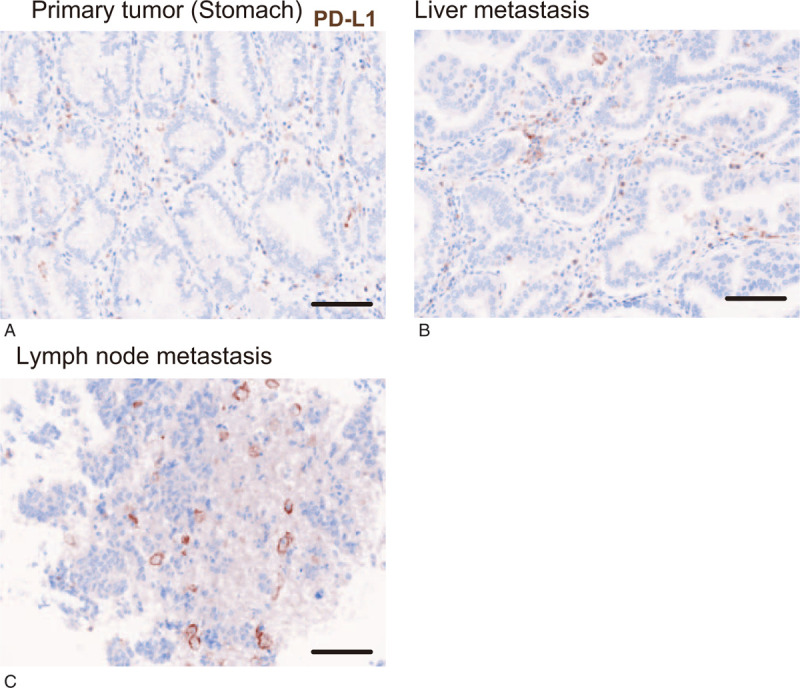
Representative images showing expression of PD-L1 (brown) in the primary tumor (A), liver metastatic tumor (B), and metastatic lymph node (C). magnification, ×200. Scale bar, 100 μm.

We analyzed 3 FFPE specimens that were available for examination: one was a primary tumor of the stomach that were resected in September 2013 (Fig. 1a, 2a), the second was a liver metastasis accompanied by abscess during SP therapy as first-line treatment (Fig. 1b, 2b), and the third was a lymph node metastasis with HPD after nivolumab therapy as third-line treatment (Fig. 1e, 2c). Using these specimens, tumor-infiltrating immune cells were analyzed with multiple immunohistochemical staining in Figure 2. In the primary tumor, infiltration of Th cells (CD3^+^CD8^−^FOXP3^−^), Treg cells (CD3^+^CD8^−^FOXP3^+^), CTLs (CD3^+^CD8^+^), and PD-L1-negative macrophages (CD3^−^CD163^+^PD-L1^−^) was found in the stroma surrounding the AE1/AE3-positive gastric cancer cells (Fig. 2a). Similarly, infiltration of these immune cells was also observed in the liver metastatic tumor (Fig. 2b). On the other hand, in the lymph node metastasis that showed accelerated tumor growth, a large number of PD-L1-positive macrophages (CD3^−^CD163^+^PD-L1^+^) were detected, whereas CTLs, Treg cells, and Th cells had almost disappeared (Fig. 2c). Figure 3 shows the percentages of immune cells that infiltrated in these 3 sections. Compared with the other specimens, CTLs and Th cells were significantly decreased in metastatic lymph nodes. Treg cells were almost undetectable in both liver and lymph node metastases. In contrast, PD-L1-positive macrophages were significantly increased in HPD lesion (Fig. 3). The percentage of PD-L1-negative macrophages did not change consistently. Furthermore, we examined PD-L1 expression in gastric cancer cells in the specimens (Fig. 4). Gastric cancer cells did not show PD-L1 expression in any specimens (PD-L1 expression: 0%), and PD-L1-positive cells were found only in stromal immune cells.

## 5 Discussion

In the present study, the distribution of immune cells in sequential specimens before and after administration of anti-PD-1 therapy was examined in a patient who presented clinically with HPD after anti-PD-1 therapy. Compared with the primary gastric tumor, a significant increase in PD-L1-positive macrophages and decreases in CTLs, Th cells, and Treg cells were observed in lymph node metastasis after anti-PD-1 therapy.

Although the biological mechanisms of HPD are not clear, several factors have been linked to HPD in recent studies in which next-generation sequencing of clinical samples and in vitro analyses were performed. Regarding extrinsic factors, infiltration and activation of M2 macrophages and Treg cells in tumor tissue and an increase in highly differentiated CD28^−^CD27^−^CD4^+^ T cells in peripheral blood have been identified in patients with HPD.^[11–13]^ Th2 cytokines such as interleukin-4 and interleukin-13 induce differentiation from monocytes into M2 macrophages, which possess protumorigenic properties in tumor tissue. Previous clinical studies reported that the presence of M2 macrophages is correlated with poor prognosis in various cancers. A recent study of NSCLC patients treated with ICIs demonstrated the infiltration of CD163^+^CD33^+^PD-L1^+^ M2-like clustered epithelial macrophages in patients with HPD but not those without HPD.^[11]^ In a mouse xenograft model derived from HPD tumor samples, M2-like macrophages increased following anti-PD-1 antibody treatment, with a concomitant large increase in tumor progression.^[11]^ The authors speculated that binding of the macrophage-FcR (CD32B) and the Fc domain of the anti-PD-1 antibody may functionally reprogram M2 macrophages into a more invasive protumorigenic phenotype.^[11]^ On the other hand, Treg cells play an important role in regulating anti-tumor T cell responses through suppressive activity on effector T cells in the tumor microenvironment. PD-1 molecules are expressed on Treg cells, and thus, activity of Treg cells could be influenced by the anti-PD-1 antibody. A recent study that compared gastric cancer tissue samples before and after anti-PD-1 antibody therapy showed that anti-PD-1 antibody treatment markedly increased tumor-infiltrating proliferative Treg cells in HPD patients, but decreased these cells in non-HPD patients.^[12]^ PD-1 blockade significantly enhanced the suppressive activity of Treg cells in vitro and in mice.^[12]^ An increased number of activated Treg cells in peripheral blood was also observed after 1 cycle of anti-PD-1 therapy and at the time of disease progression in retrospective studies of melanoma and gastric cancer patients.^[14,15]^ From these findings, M2 macrophages and Treg cells activated by the anti-PD-1 antibody may be part of the cause of HPD. Consistent with the previous report referring to M2 macrophages in NSCLC patients, a significant increase in CD163^+^PD-L1^+^ M2 macrophages and a decrease in CTLs and Th cells in HPD tissue were observed in the present study. No increase in Treg cells was observed in HPD tissue. This may be caused by the difficulty in distinguishing between high and weak expression of FoxP3 with immunohistochemistry analysis. The present study may have included non-Treg cells that weakly expressed FoxP3 in addition to effector Treg cells that highly expressed FoxP3 in the analysis. In another study of 45 NSCLC patients who received ICIs, 21 patients with an increase in the CD28^−^CD27^−^CD4^+^ T cell subset after 1 cycle of ICIs had no response, and all experienced disease progression.^[13]^ In addition, patients with HPD had a lower number of CD28^−^CD27^−^CD4^+^ T cells in baseline peripheral blood compared to those who progressed on ICIs but did not develop HPD.^[13]^ Taken together, the CD4 subset may be a potential biomarker for identifying patients who are at risk of HPD on ICI treatment. However, the behavior of CD4^+^ cells in tumor tissue has not been investigated.

Regarding intrinsic factors of HPD, in some large retrospective studies including a variety of tumor types, *MDM2* amplification and *EGFR* mutation are highly prevalent in patients with HPD.^[16,17]^*MDM2/EGFR* genomic aberrations drive or predispose certain individuals to developing HPD. In another meta-analysis of 9 articles that included 217 HPD patients and 1519 cancer patients treated with anti-PD-1/PD-L1 therapy, positive PD-L1 expression on tumor cells was inversely correlated with HPD.^[18]^ PD-L1 expression on tumor cells in our case was negative, which is consistent with the report.

In conclusion, few report has shown detailed immune cell phenotyping with HPD tissue in AGC patients with anti-PD-1 antibody treatment. We conducted multiplex immunohistochemical staining and quantitative analysis with a metastatic lymph node in a patient with HPD. This is the first report to confirm a significant increase in M2 macrophages and a decrease in CTLs and Th cells in HPD tissue of an AGC patient. PD-L1 positive M2 macrophages were overrepresented in the metastatic lymph node that showed HPD in the present study, which may have led to tumor growth. The present study is based on the results from only 1 case and does not prove the direct interaction between anti-PD-1antibody and PD-L1 positive macrophages. It also assessed the cancer microenvironment of different primary or metastatic tissues, thus it may need to take into account of the native differences in microenvironment between the tissues. Although the mechanism of M2 macrophage activation by the anti-PD-1 antibody has not been fully examined, the present study may provide insight into the mechanism of HPD.

## Author contributions

**Conceptualization:** Kenji Tsuchihashi, Eishi Baba, Kyoko Yamaguchi.

**Data curation:** Kyoko Yamaguchi.

**Formal analysis:** Kyoko Yamaguchi.

**Funding acquisition:** Koichi Akashi, Eishi Baba.

**Investigation:** Kyoko Yamaguchi.

**Methodology:** Kyoko Yamaguchi, Mamoru Ito.

**Resources:** Kunihiro Tsuji, Yosuke Kito.

**Writing – original draft:** Kyoko Yamaguchi.

**Writing – review & editing:** Kenji Tsuchihashi, Kenro Tanoue, Hirofumi Ohmura, Mamoru Ito, Taichi Isobe, Hiroshi Ariyama, Hitoshi Kusaba, Koichi Akashi, Eishi Baba.
